# Motor Unit Force Potentiation and Calcium Handling Protein Concentration in Rat Fast Muscle After Resistance Training

**DOI:** 10.3389/fphys.2021.652299

**Published:** 2021-05-14

**Authors:** Dawid Łochyński, Dominik Kaczmarek, Marcin Grześkowiak, Joanna Majerczak, Tomasz Podgórski, Jan Celichowski

**Affiliations:** ^1^Department of Neuromuscular Physiotherapy, Poznan University of Physical Education, Poznań, Poland; ^2^Department of Physiology and Biochemistry, Poznan University of Physical Education, Poznań, Poland; ^3^Department of Cardiological and Rheumatological Rehabilitation, Poznan University of Physical Education, Poznań, Poland; ^4^Department of Neurobiology, Poznan University of Physical Education, Poznań, Poland

**Keywords:** strength training, excitation contraction coupling, calcium release, muscle fibers, fatigue, window of opportunity

## Abstract

Post-tetanic potentiation (PTP) of force depends on intramuscular Ca^2+^ levels and sensitivity and may be affected by fatigue. The aim of this study was to determine the ability of isolated fast fatigue-resistant (FR) and fast-fatigable (FF) motor units (MUs) to potentiate force evoked with single and 40-Hz electrical stimulation after 5 weeks of voluntary weight-lifting training. Tetanic contractions evoked by gradually increasing (10–150 Hz) stimulation frequency served as conditioning stimulation. Additionally, the concentration of myosin light chain kinase and proteins engaged in calcium handling was measured in rat fast medial gastrocnemius muscle. After the training, the potentiation of twitch force and peak rate of force development was increased in FF but not FR MUs. Force potentiation of 40-Hz tetanic contractions was increased in both fast MU types. After the training, the twitch duration of FR MUs was decreased, and FF MUs were less prone to high-frequency fatigue during conditioning stimulation. Muscle concentration of triadin was increased, whereas concentrations of ryanodine receptor 1, junctin, FKBP12, sarcoplasmic reticulum calcium ATPase 1, parvalbumin, myosin light chain kinase, and actomyosin adenosine triphosphatase content were not modified. After short-term resistance training, the twitch contraction time and twitch:tetanus force ratio of FR MUs are decreased, and PTP ability is not changed. However, PTP capacity is increased in response to submaximal activation. In FF MUs increase in PTP ability coexists with lesser fatigability. Further work is required to find out if the increase in triadin concentration has any impact on the observed contractile response.

## Introduction

Post-tetanic potentiation (PTP) is expressed as an enhancement of twitch contraction force evoked after preceding high-intensity muscular contractile activity. In humans (Hamada et al., 2000) and other mammals (Moore and Stull, 1984), increased force potentiation is observed in muscles with a higher percentage of fast fiber types and shorter twitch contraction time. PTP might be mediated through increased phosphorylation of myosin regulatory light chain (RLC) along with increased sensitivity of cellular contractile proteins to Ca^2+^ (Stull et al., 2011). Currently, there is limited knowledge on the potential effects of resistance training on PTP and contractile modifications. In only one report, increased postactivation potentiation with concomitant reduction of twitch force and twitch:maximum voluntary force ratio was found in humans after several weeks of low-intensity resistance training with vascular occlusion (Moore et al., 2004). Also, it is largely unknown whether resistance training modifies the concentration of proteins in the skeletal muscle, which regulates force potentiation ability.

Previously, we noted that short-term resistance training affects twitch contractile parameters in fast fatigue-resistant (FR) but not fast-fatigable (FF) motor units (MUs). In the former, decrease in duration of twitch contraction and relaxation times and reduction of twitch:maximum tetanus force ratio was present. This suggests a decrease in the amount of Ca^2+^ released during the twitch contraction. Ca^2+^ release may be affected by modifications in triadin and junctin, the major transmembrane proteins, which form a complex with the ryanodine receptor (Ca^2+^ release channel) in skeletal muscle sarcoplasmic reticulum (Beard et al., 2009). For instance, triadin negatively regulates RyR function by suppression of the depolarization-induced SR Ca^2+^ release (Rezgui et al., 2005), and junctin mediates signals between luminal Ca^2+^, skeletal muscle calsequestrin, and ryanodine receptors (Wei et al., 2009). Moreover, reduction of the twitch duration and amplitude may be related to shortening of the Ca^2+^ transient. This can be caused by an increase in the concentration of Ca^2+^ binding and transporting proteins, such as parvalbumin and SR Ca^2+^ pump in the muscle fibers (Berchtold et al., 2000).

Short-term training is a heavy conditioning stimulus that appears to increase central nervous system excitation and MU activation leading to an increase in muscle force (Vecchio et al., 2019). The frequency and the time of muscle fiber activation determines the amount of Ca^2+^ released from the sarcoplasmic reticulum (SR) (Baylor and Hollingworth, 2012). We suppose that, at the early stage of resistance training, increased firing activity of particular MUs during each weight-lifting repetition results in high rates of Ca^2+^ cytosolic oscillations. In turn, a substantial amount of Ca^2+^ is released from SR during each lifting attempt, which results in large elevation of Ca^2+^ above its native levels. This could override the natural Ca^2+^-dependent inactivation of Ca^2+^ release from the SR seen during closely spaced contractions (Hollingworth et al., 1996). Therefore, it can be supposed that several weeks of training-related, repetitive, large SR Ca^2+^ cytoplasmic fluxes due to short high-frequency bursts of MU activity would ultimately result in adjustments in concentrations of specific Ca^2+^ SR release and sequestration proteins in the muscle. This could be responsible for reduction of twitch contraction duration and amplitude observed after resistance training and, in turn, result in suppression of full twitch PTP ability of MUs.

The aim of the present study was to characterize twitch potentiation of fast MUs and to determine muscle concentrations of regulatory proteins involved in SR calcium release and force potentiation after short-term, voluntary resistance training. It was supposed that twitch potentiation ability may be suppressed in FR MUs as their twitch duration and force are reduced after the training. It was assumed that, in FF units, the twitch potentiation is enhanced as their contractile parameters are not modified after the short-term resistance training (Łochyński et al., 2016). We also hypothesized that there would be modifications in muscle protein content, known to act toward the suppression of calcium release from SR, such as an increase in muscle triadin content (Rezgui et al., 2005), as well as toward the enhancement of force potentiation, i.e., an increase in skeletal muscle myosin light chain kinase (skMLCK) concentration/activity (Ryder et al., 2007).

## Materials and Methods

### Animal Care and Training Procedures

The data reported in this study were collected from rats used in the previous work (Łochyński et al., 2016) and two additional animals (one trained and one control rat). Overall, 23 male Wistar rats (15 weeks old), randomly assigned to either the control sedentary group (C; *n* = 11) or the weight-lifting group (WL; *n* = 11), were involved. One rat from the WL group did not accomplish the training program and was excluded. Animals were treated equally and housed in standard laboratory cages in an animal house with the humidity at 55 ± 10%, temperature at 22 ± 2°C, and 12-h light/dark cycle. Animal care and experimental procedures were accepted by the Local Ethics Committee for Animal Research and performed in accordance with the European Union guidelines and Polish Law on Protection of Animals and Protection of Animal Health and Control of Infectious Diseases of Animals.

The voluntary weight-lifting training program (the exercise apparatus, learning, intensity, frequency, and duration) and procedures (the adaptation to exercise, the feeding schedule, and control of training) are described in detail in the preceding paper (Łochyński et al., 2016). In brief, the rats lifted weights put on their shoulders in a squat-type manner while reaching for food (a standard commercial chow, Labofeed B, Poland) in a custom-made apparatus. Rats initially learned to perform exercises during 2 weeks of adaptation and then performed weight-lifting training, which consisted of two 30-min sessions (at 8 am and 3 pm) a day performed 5 days a week for 5 weeks. At the same time, their control counterparts were moved to separate cages and had free access to food. Any difference in the amount of food consumed between both groups of animals was registered and balanced each day during the entire study period. In the evening of each exercise day, rats were supplemented with an additional 12 g of chow/animal. In each training week, rats were supplemented with 60 g of chow after the fifth day of exercise, which lasted until the start of the next week. Therefore, the schedule of feeding and the amount of food consumed was the same throughout the study. During the leisure time, both groups were restricted to natural physical activity in cages, and the only difference was that, when WL rats performed voluntary progressive weight-lifting exercises, the C rats were engaged in eating activity. After the weight-lifting training program, the body mass as well as absolute and normalized muscle weights did not differ between the groups (Table 1).

**TABLE 1:** Medial gastrocnemius muscle characteristics in the studied groups of animals.

	**C (*n* = 11)**	**WL (*n* = 11)**	**Calculated statistics**	***p***
Body mass (g)	452.244.3	434.235.0	*t*(20) = −1.1	0.302
MG muscle mass (g)	1.050.12	1.040.07	*t*(20) = −0.2	0.880
MG muscle/body mass (%)	0.230.04	0.240.02	*t*(20) = 0.6	0.588

*Mean ± SD are presented. MG, medial gastrocnemius; *n*, the number of animals; *p*, probability value.*

During lifting, rats executed ankle and knee extension and flexion movements. The apparatus was fitted to the individual sizes of the animals. At the beginning of each week, the one repetition maximum load that could be lifted by a rat was determined. The exercise load grew between the first and fourth week from 70% up to 85% of one repetition maximum and was maintained at this level in the fifth week of training. Over the exercise period (between the first and last weeks of training) the mean absolute 1 RM load mass increased from 743.1 ± 179.2 g to 1158.3 ± 283.6 g [*p* < 0.001], and 1 RM load mass relative to body mass increased from 160.0 ± 18.9% to 317.1 ± 70.6% [*p* < 0.001]. The information on changes in exercise determinants (e.g., work and power performed during lifting) for the entire training period is detailed in the previous work (Łochyński et al., 2016).

### Surgical and Electrophysiological Procedures

Within 48–72 h after the completion of the exercise program, animals were anesthetized by intraperitoneal injection of pentobarbital sodium (initial dose 60 mg/kg). Anesthesia was maintained by additional doses of 10 mg/kg supplemented approximately every hour and controlled by observation of pinna and limb withdrawal reflexes. Administration of a lethal dose of pentobarbital (180 mg/kg) terminated the experiments.

As described previously (Łochyński et al., 2016), in six rats from the WL group and seven rats from the C group, the L4 and L5 ventral roots were exposed by laminectomy. The left medial gastrocnemius (MG) was dissected from the surrounding tissues and connected via a low-compliance surgical thread to an inductive force transducer (deflection sensitivity of 100 μm per 100 mN). The muscle was stretched up to a passive force of 100 mN, at which the majority of its MUs generate maximum twitch amplitudes (Celichowski and Grottel, 1992). The ventral roots, cut and split into the thinnest possible filaments, were stimulated by rectangular electrical pulses of 0.1 ms duration and variable voltage, up to 0.5 V (model S88; Grass Instrument). The procedure of MU isolation was achieved by finely adjusting the stimulus amplitude around the threshold value until the action potential was recorded with two non-insulated silver wires (300 μm in diameter) inserted in the central part of the muscle belly, and the twitch forces were of the all-or-none type. During the entire experiment, the animal’s core temperature was automatically maintained at a constant level of 37 ± 1°C by a closed-loop temperature controller.

### Testing Protocol, Data Analyses, and MU Classification

The following stimulation protocol was administered: (1) five pulses at 1 Hz (non-potentiated twitches); (2) a 500-ms train at 40 Hz (the unfused tetanus); (3) a 300-ms train at 150 Hz and successive 500-ms trains at 10, 20, 30, 40, 50, 60, 75, 100, and 150 Hz [conditioning stimulation (Łochyński et al., 2007)]; and (4) five pulses at 1 Hz (potentiated twitches). Particular steps and stimulations within step 3 of the protocol were separated by 10-s intervals. The standard fatigue test, i.e., 325-ms trains at 40 Hz delivered one time per second for 3 min, ended the stimulation (step 5). The force was measured under isometric conditions. For each MU, the twitch peak force (TwF), peak rate of force development (RFD), and contraction time (CT) were measured for the averaged initial twitches evoked at step 1 and potentiated twitches evoked at step 5 of the protocol. The potentiation capacity for submaximal contraction was measured by comparing peak force of the 40-Hz tetanic contraction of step 2 of the protocol with the first contraction of step 5 of the protocol. The peak forces of the 150-Hz tetanic contractions at the beginning and end of conditioning stimulation were measured to verify the development of potential fatigue. The twitch:tetanus force ratio was calculated as the ratio of non-conditioned twitch and 150-Hz tetanus at baseline. The PTP was calculated as the peak force ratio of the potentiated to non-potentiated initial twitch. MUs with a sag in force profile in the unfused tetanus evoked at 40 Hz were studied and classified as the FF type when the fatigue index was below 0.5 or as the FR type when the index exceeded 0.5 (Łochyński et al., 2007). Peak forces of 40 Hz tetani were measured every 5 s during the initial 20 s and later every 10 s during the fatigue test. To compare force profiles of all fast MUs, they were expressed as percentages of the peak force of the second tetanus within the test (Łochyński et al., 2016).

### Protein Extraction

Analyses of muscle protein content were performed on the left MG. Muscles were carefully excised, and any visible connective or fat tissues were removed. Muscle samples were weighed, placed in cryogenic vials (NUNC/Thermo Fisher Scientific), immediately frozen in liquid nitrogen, and then kept at −80°C. On the day of the biochemical analyses, the muscle samples were subsequently dissolved and homogenized in buffer saline solution (PBS, with tissue to buffer ratio 1:9 according to the ELISA kit procedures). Samples were homogenized using a VWR VDI 12 disperser homogenizer (Singapore) with 28,000–30,000 rpm, centrifuged, and finally the supernatant was removed and kept in Eppendorf tubes at 4°C until assayed.

### Biochemical Analyses

The specific Ca^2+^ SR release and sequestration protein content was determined in MG using commercially available enzyme-linked immunosorbent assay (ELISA) kits (Shanghai Sunred Biological Technology Co., Ltd., Shanghai, China): ryanodine receptor 1 (RYR1, Cat. No 201-11-1614), triadin (TRDN, Cat. No 201-11-2669), junctin (Cat. No 201-11-5543) and FK506 binding protein 12 (FKBP12, Cat. No 201-11-5320), sarcoplasmic/endoplasmic reticulum calcium ATPase 1 (ATP2A1/SERCA1, Cat. No 201-11-2677) isoform specific to the fast-twitch skeletal muscle, and parvalbumin (PVALB, Cat. No 201-11-1444). Also, the muscle content of proteins involved in skeletal muscle force potentiation, that is, skeletal muscle myosin light chain kinase (skMLCK, Cat. No 201-11-0311) and actomyosin adenosine triphosphatase (amATPase, Cat. No 201-11-2243), was estimated. A multimode microplate reader (Synergy 2 SIAFRT, BioTek, United States) was used to measure absorbance at 450 nm. The concentrations of proteins were expressed as pg in 1 ml of supernatant for junctin, and in ng in 1 ml of supernatant for the other studied proteins.

### Statistical Analyses

After testing the normality of the data with the Shapiro–Wilk test, twitch contraction parameters and 40-Hz tetanic force were subjected to a two-way analysis of variance having two levels of group (untrained and weight-lifting trained) and two levels of contraction (non-potentiated and potentiated). Multiple pairwise *post hoc* tests with the two *a priori* hypotheses were conducted using Bonferroni adjusted alpha levels of 0.025 per test (05/2). The PTP ratios and concentrations of muscle proteins in both groups were compared with the parametric Student *t*-test (for normal distribution and equal variances of the data) or non-parametric Mann–Whitney *U* test (for non-normal distribution and non-equal variances of the data). A paired *t*-test was used to compare the absolute and relative exercise load at the beginning and end of the training period. Pearson product moment correlation or Spearman rank correlation coefficients (depending on data normality) were calculated to express the relationship between MUs’ twitch contraction time and the amount of relative force potentiation. Linear regressions were also calculated. For the best statistical evaluation of force profile during the fatigue test, its course was divided into the three fairly linear time zones: 0–20 s, 30–70 s, and 80–180 s, and two-way repeated-measures analysis of variance (ANOVA) was performed with group (WL vs. C) and time as the factors. All effects were considered statistically significant at α < 0.05. Eta square, Cohen’s d, or *R* (for correlations) values were calculated to express the size of effects.

## Results

### The Effect of Weight-Lifting Training on the Parameters of Potentiated Twitches in Whole Population of Fast MUs

**FIGURE 1:**
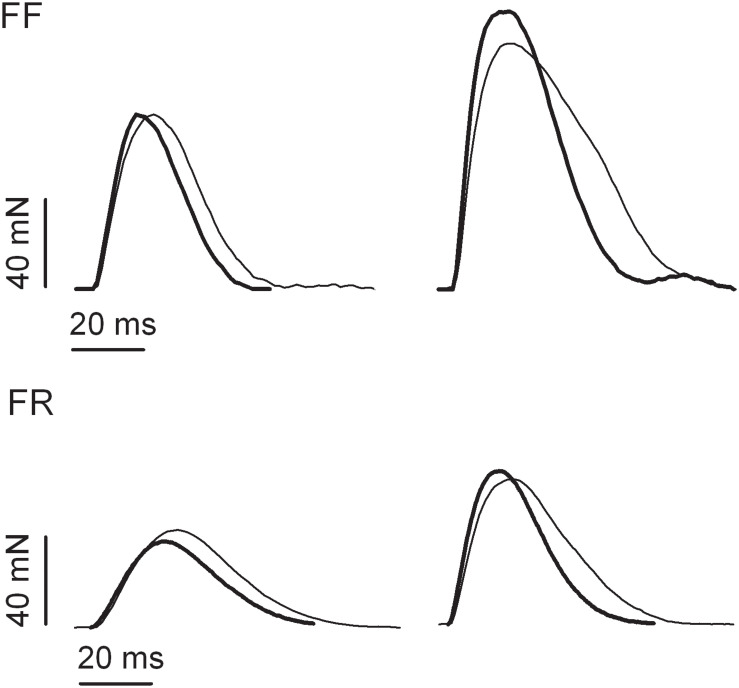
Examples of isometric twitch responses of fast fatigable (FF) and fast fatigue resistant (FR) motor units of medial gastrocnemius muscle obtained from untrained (thin lines) and resistance-trained animals (thick lines).

**FIGURE 2:**
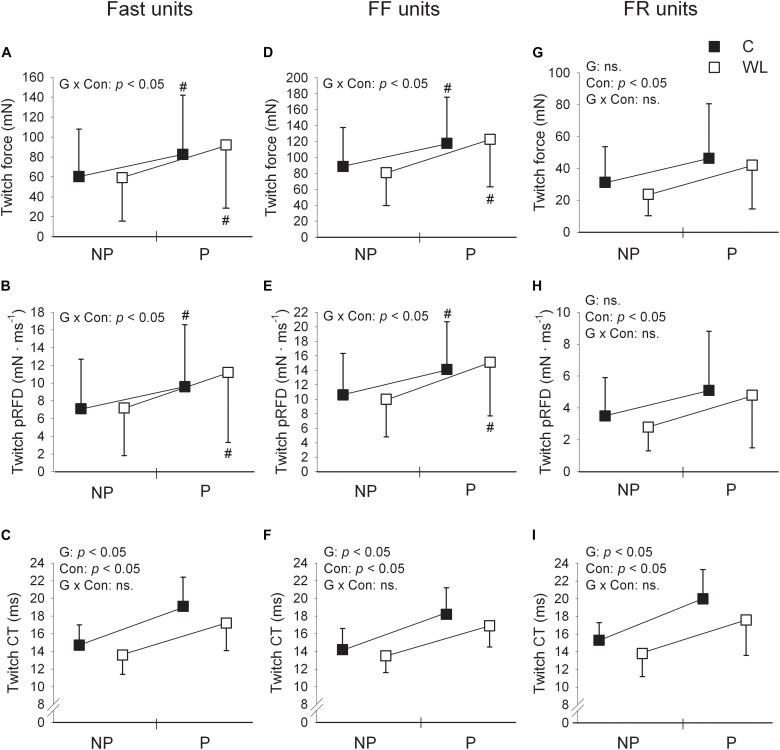
Comparison of fast **(A–C)**, FF **(D–F)**, and FR **(G–I)** motor units’ contractile parameters for pre and post tetanic conditioning stimulation measurements in control (C) and weight-lifting trained (WL) animals. Data are presented as mean values and standard deviations. FF, fast fatigable; FR, fast fatigue resistant; pRFD, peak rate of force development; CT, contraction time; NP, non-potentiated; P, potentiated. The impact of factors G, group (control vs. weight lifting); Con, contraction (twitch non-potentiated vs. potentiated state); and G × Con, interaction between the group and contraction were expressed as significant (*p* < 0.05) or not significant (ns.) *p* values, presented within panels. *Post hoc* analysis results: # = significant difference between pre and post measurements. Non-potentiated twitch force and contraction time values are from the previous study (Łochyński et al., 2016) and are included for comparison. Note much substantial shortening of the contraction time of the potentiated twitch (much higher partial eta squared values for the ANOVA’s effect of group, Table 2) in FR [**(I)**, large effect size] than FF [**(F)**, small effect size] motor units. In addition, when control and weight-lifting rats were analyzed with two-sided *post hoc* comparisons, effect sizes expressing the magnitude of decrease in the contraction time of potentiated twitches were also higher in FR (Cohen’s d = 0.65) than FF (Cohen’s d = 0.48) motor units. These observations coincided with increased potentiation of twitch force and pRFD in FF **(D,E)** but not FR **(G,H)** motor units.

For the entire population of fast MUs sampled from the medial gastrocnemius muscle, training increased potentiation of the TwF and peak RFD (significant effect of group × contraction interaction, Figures 1, 2A,B and Table 2). The TwF and peak RFD ratios of potentiated to non-potentiated twitches were increased (Table 3).

**TABLE 2:** Summary ANOVA results on effects of training on twitch post-tetanic potentiation in fast MUs.

	**Twitch force**					**Twitch pRFD**						**Twitch CT**				
**Source**	***F_(1,168)_***	***p***		η**_*p*_^2^**		***F_(1,168)_***		***p***		η**_*p*_^2^**		***F_(1,168)_***		***p***		η**_*p*_^2^**

***Whole population of fast motor units***																
Group												20.8		<0.001		0.110
Contraction												331.7		<0.001		0.664
Group × con	12.0	0.002		0.067		12.0		0.001		0.066		5.4		0.137		0.013

	**Twitch force**						**Twitch pRFD**						**Twitch CT**			
**Source**	***F_(1,95)_***		***p***		η**_*p*_^2^**		***F_(1,95)_***		***p***		η**_*p*_^2^**		***F_(1,95)_***		***p***	η**_*p*_^2^**

***FF motor units***																
Group													4.4		0.039	0.044
Contraction													379.1		<0.001	0.800
Group × con	9.8		0.002		0.076		8.3		0.005		0.080		2.8		0.098	0.029

	**Twitch force**					**Twitch pRFD**							**Twitch CT**			
**Source**	***F_(1,71)_***	***p***		η**_*p*_^2^**		***F_(1,71)_***			***p***		η**_*p*_^2^**	***F_(1,71)_***		***p***		η**_*p*_^2^**

***FR motor units***																
Group	1.09	0.300		0.015		0.720			0.399		0.010	9.1			0.004	0.114
Contraction	84.3	<0.001		0.543		67.9			<0.001		0.489	182.2			<0.001	0.720
Group × con	0.72	0.398		0.010		1.01			0.300		0.015	1.93			0.169	0.026

*Group (control vs. weight lifting), Contraction (twitch non-potentiated vs. potentiated state), pRFD, peak rate of force development; CT, contraction time; (ηp^2^), = partial eta squared (effect size); con, contraction; FF, fast fatigable; FR, fast resistant.*

**TABLE 3:** Post tetanic potentiation (PTP) ratios of the potentiated twitch to the unpotentiated twitch in fast motor units of untrained and weight-lifting trained animals.

	**C (*n* = 6)**	**WL (*n* = 7)**
***F***	***(n = 75)***	***(n = 95)***

TwF	1.4 ± 30.31	1.6 ± 30.36^§^
TwRFD	1.4 ± 10.31	1.60 ± 0.39^§^
CT	1.30 ± 0.16	1.27 ± 0.17

***FF***	***(n = 38)***	***(n = 59)***

TwF_*PTP*_	1.40 ± 0.23	1.57 ± 0.32^§^
TwRFD	1.41 ± 0.27	1.57 ± 0.34^§^
CT	1.29 ± 0.16	1.26 ± 0.13

***FR***	***(n = 37)***	***(n = 36)***

TwF	1.46 ± 0.32	1.73 ± 0.40^§^
TwRFD	1.41 ± 0.33	1.65 ± 0.47^§^
CT	1.31 ± 0.16	1.29 ± 0.21

*Data are presented as means ± standard deviations. C, control rats; WL, weight-lifting rats; F, fast; FF, fast fatigable; FR, fast fatigue resistant; TwF, twitch force; RFD, peak rate of force development; CT, contraction time. ^§^, significant difference between corresponding PTP ratios in C and WL groups (*p* < 0.05).*

In both WL and C, group conditioning stimulation induced a similar (non-significant effect of group × contraction interaction, Figures 1, 2C and Table 2) increase in the CT due to the twitch contraction potentiation. In the WL group, the twitch CT was decreased (in both the potentiated and non-potentiated state combined) as compared with the C group (significant effect of group, Figure 2C and Table 2). The contraction time ratio of potentiated to non-potentiated twitches was not changed (Table 3).

### The Effect of Weight-Lifting Training on the Parameters of Potentiated Twitches in FF MUs

Training significantly increased the potentiation of TwF and peak RFD (significant effect of group × contraction interaction, Figures 1, 2D,E and Table 2). The TwF and peak RFD ratios of potentiated to non-potentiated twitches were increased (Table 3).

In both WL and C groups, conditioning stimulation induced similar (non-significant effect of group × contraction interaction) increase in the CT due to the twitch contraction potentiation (Figures 1, 2F and Table 2). However, in the WL group, the twitch CT was decreased (in both the potentiated and non-potentiated state combined) as compared with the C group (significant effect of group, Figure 2F and Table 2). The CT ratio of potentiated to non-potentiated twitches was not changed (Table 3).

### The Effect of Weight-Lifting Training on the Parameters of Potentiated Twitches in FR MUs

In FR MUs, training did not affect either the twitch contraction force or peak RFD (non-significant effect of group × contraction interaction, Figures 1, 2G,H and Table 2). However, the twitch contraction force and peak RFD ratios of potentiated to non-potentiated twitches were increased after the training (Table 3).

In C and WL groups, conditioning stimulation induced a similar (non-significant effect of group × contraction interaction) increase in the contraction time (Figures 1, 2I and Table 2) due to the twitch contraction potentiation. Nevertheless, in the WL group, the twitch CT was decreased (in both the potentiated and non-potentiated state combined) as compared with the C group (significant effect of group, Figure 2I and Table 2). The CT ratio of potentiated to non-potentiated twitches was not changed (Table 3).

### The Effect of Weight-Lifting Training on the Relationship Between the Non-potentiated Twitch Parameters, Twitch:Tetanus Force Ratio, and Force Potentiation

**FIGURE 3:**
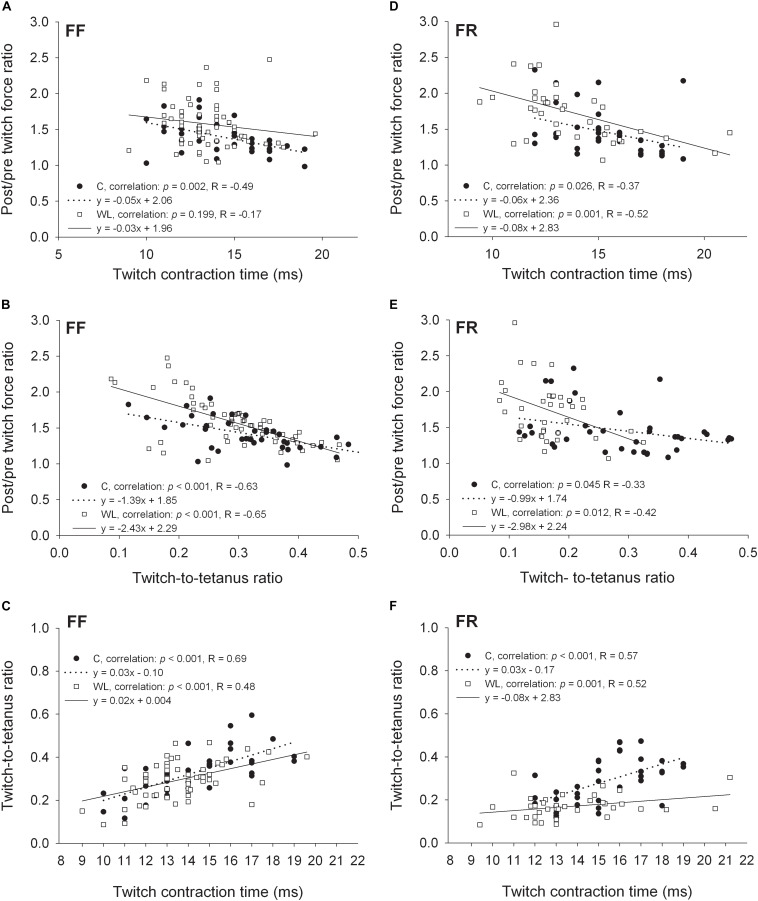
Relationships between the non-potentiated twitch contraction times, twitch:tetanus force ratios, and force ratios of the potentiated to non-potentiated twitches for FF **(A–C)** and FR **(D–F)** motor units. C, control group; WL, weight lifting group.

In FF and FR MUs of non-trained animals, low negative correlations were observed between the non-potentiated twitch contraction time and the amount of relative force potentiation. After the training, this relationship was absent in FF (Figure 3A) and maintained in FR MUs (Figure 3D). FF MUs of trained as well as FR MUs of trained and untrained animals with shorter twitch contraction durations were able to potentiate force slightly more than those with longer durations (Figures 3A,D). In FR MUs, the slope of the relationship was similar in the trained and untrained animals (Figure 3D). On the other hand, in FF MUs of trained animals, differently than in control animals, PTP capacity became similar in FF MUs with shorter and longer contraction duration (loss of correlation, Figure 3A).

In the trained and untrained animals, moderate and low negative correlations were present between the non-potentiated twitch-to-tetanus force ratios and the amount of relative force potentiation in FF (Figure 3B) and FR (Figure 3E) MUs, respectively. FF and FR MUs with the lower twitch:tetanus force ratios were able to potentiate force more than those with the higher ratios. However, in both MU types (especially in FR units) the slopes of these relationships were steeper in the trained than untrained animals. Also, FR MUs of trained rats had lower twitch:tetanus force ratios than those of untrained rats (*p* < 0.001, Mann Whitney rank sum test, leftward migration of the values along the abscissa), and FF units did not (*p* = 0.115, Student’s *t*-test).

Moderate positive correlations between the twitch contraction duration and twitch:tetanus force ratio were observed in fast MUs of trained and untrained animals. In FF units, the steepness of the regression line was similar (Figure 3C), and in FR MUs, lower steepness was noted after the training (Figure 3F). Thus, generally MUs with longer twitch contraction durations had higher twitch:tetanus force ratios than units with shorter durations. Nevertheless, this relationship was much less pronounced in FR units after the training as twitch duration had a lower effect on the twitch:tetanus force ratio (Figure 3F).

### The Effect of Weight-Lifting Training on Force Potentiation During Tetanic Stimulation

**FIGURE 4:**
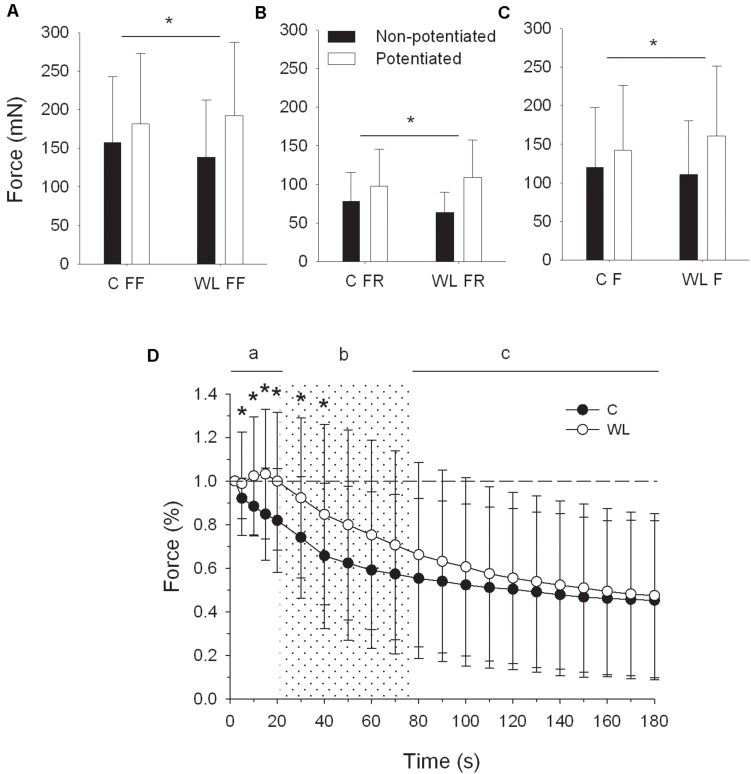
Peak force potentiation of 40 Hz tetanus after resistance training **(A–C)**, and force profile of 40 Hz tetanus during repeated fatiguing stimulation **(D)**. **(A–C)**, * denotes significant differences (*p* < 0.05, *post hoc* comparisons) in force augmentation between trained and untrained rats. **(D)**, * denotes significant differences (*p* < 0.05, *post hoc* comparisons) between forces of trained and untrained rats for the whole population of fast motor units. Note that force was initially slightly potentiated in trained rats and declined steadily in untrained animals. C, control group; WL, weight lifting group; FR, fast fatigue resistant motor units; FF, fast fatigable motor units; F, whole population of fast motor units.

After training, the potentiation of peak force of 40 Hz tetanic contractions was enhanced in FF [significant effect of group × contraction interaction, *F*_(__1_,_94__)_ = 13.141; *p* < 0.001, Figure 4A], FR [significant effect of group × contraction interaction, *F*_(__1_,_67__)_ = 12.622; *p* = 0.001, Figure 4B], and in the whole sample (FF and FR) of fast MUs [significant effect of group × contraction interaction *F*_(__1_,_163__)_ = 26.434; *p* < 0.001, Figure 4C].

In fast MUs of WL rats (Figure 4D), the peak force of 40-Hz tetanic contractions evoked by a repetitive 40-Hz stimulation was initially potentiated from the fifth to the 10th second and then declined to the initial force at the 20th second of stimulation. In the same time, the force of untrained animals decreased steadily [significant effect of group × time interaction: *F*_(__3_,_477__)_ = 7.088; Greenhouse–Geisser *p* = 0.004, time frame “a” on Figure 4D]. Then, the force of WL rats declined more (significant effect of group and time interaction: *F*_(4_,_636)_ = 3.770; Greenhouse–Geisser *p* = 0.033) but still was maintained at a higher level, up to the 40th second of stimulation (*post hoc* comparisons, time frame “b” on Figure 4A). Finally, force declined more in WL rats between the 80th and 180th seconds (significant interaction effect between group and time: *F*_(10_,_1590)_ = 4.313; Greenhouse–Geisser *p* = 0.026) to reach a similar level for both groups at the end of the repeated stimulation (time frame “c” on Figure 4D).

### The Effect of Weight-Lifting Training on the High-Frequency Tetanic Force Response During Conditioning Stimulation

In FF units, the peak force of 150 Hz tetanus evoked at the end of conditioning stimulation (C: 241.4 mN, WL: 263.4 mN) was slightly decreased with respect to that evoked at the beginning of stimulation (C: 261.4 mN, WL: 272.9 mN). After the training, this decrease was less pronounced [significant effect of group × contraction interaction, *F*_(__1__–__95__)_ = 5.5, *p* = 0.022]. In FR units, the peak force evoked by high-frequency stimulation (C: 103.1 mN, WL: 137.4 mN at the beginning; C: 108.4 mN, WL: 143.3 mN at the end) was not decreased [non-significant effect of group × contraction interaction, *F*_(__1__–__71__)_ = 0.2, *p* = 0.663].

### The Effect of Weight-Lifting Training on the Protein Content in Medial Gastrocnemius

**FIGURE 5:**
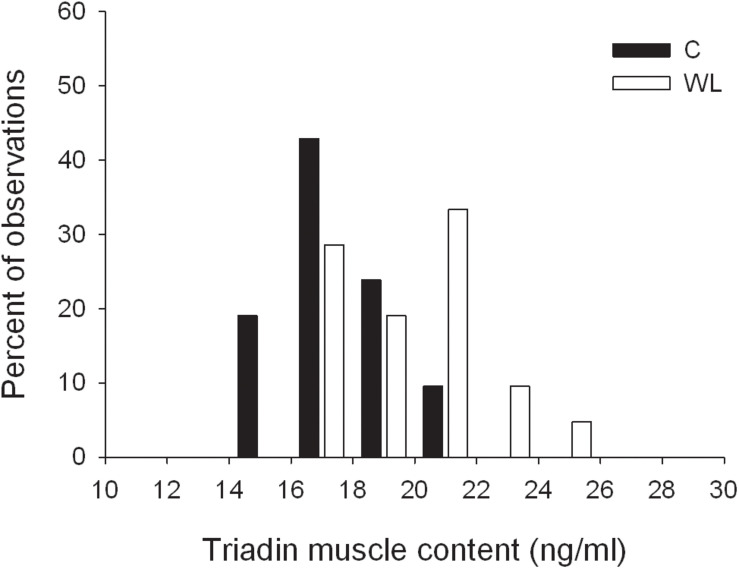
Distribution of triadin muscle concentrations in trained and untrained rats.

The weight lifting increased the level of triadin in the muscle (Table 4 and Figure 5), whereas the concentrations of other proteins involved SR Ca^2+^ release, i.e., ryanodine receptor and junctin, did not change. Similarly, concentrations of the Ca^2+^ sequestering proteins (i.e., SR calcium pump and parvalbumin) were not altered by the weight lifting (Table 4). Training had no effect on the concentration of the skMLCK and amATPase (Table 4).

**TABLE 4:** The content of proteins involved in calcium handling and in myosin light chain phosphorylation in medial gastrocnemius.

	**C (*n* = 11)**	**WL (*n* = 11)**	**Calculated statistics**	**d (LCI, UCI)**	***p* value**
**Calcium handling proteins**					
RYR1 (ng/ml)	26.0 (24.6–26.7)	24.6 (24.1–26.1)	*t*(20) = −0.6	0.3 (−2.2, 1.2)	0.528
TRDN (ng/ml)	18.9 (18.1–19.3)	20.1 (19.6–22.0)	*t*(20) = 3.4	1.5 (0.7, 3.0)	0.003
JUNCT (pg/ml)	317.0 (268.2–341.1)	274.8 (259.2–319.1)	*t*(20) = −0.9	0.4 (−54.3, 22.4)	0.396
SERCA 1 (ng/ml)	50.7 (49.8–53.1)	49.2 (45.8–56.8)	*t*(20) = −0.1	0.1 (−5.5, 4.9)	0.900
PARV (ng/ml)	138.0 (123.8–143.5)	134.1 (124.9–137.9)	*t*(20) = −0.2	0.1 (−14.9, 12.1)	0.829
FKBP12 (ng/ml)	834.9 (757.3–847.1)	830.8 (790.7–884.1)	*t*(20) = −0.5	0.2 (−86.4, 52.3)	0.613
**Proteins involved in myosin light chain phosphorylation**					
skMLCK (ng/ml)	35.8 (35.1–38.9)	33.9 (32.6–39.9)	*U* = 42.0	0.2 (−4.6, 3.0)	0.237
amATPase (ng/ml)	115.9 (110.3–128.6)	114.7 (112.3–117.5)	*t*(20) = 0.0	0.0 (−6.6, 6.7)	0.988

*Data are presented as medians (25–75% interquartile range). C = control rats; WL = weight lifting rats; d, Cohen’s effect size; LCI and UCL = the lower and the upper 95% confidence interval for difference of means; RYR1 = ryanodine receptor 1; TRDN = triadin; JUNCT = junctin; FKBP12 = FK506 binding protein 12; SERCA1 = sarcoplasmic/endoplasmic reticulum calcium ATPase 1 associated with the fast-twitch skeletal muscle; PARV = parvalbumin; skMLCK = skeletal myosin light chain kinase; amATPase = actomyosin adenosine triphosphatase.*

## Discussion

The present study showed that 5 weeks of resistance training resulted in the enhancement of twitch potentiation and peak rate of force development of FF MUs but did not affect twitch potentiation in FR MUs in rat medial gastrocnemius muscle. In the trained group, the twitch contraction duration was significantly decreased in FR and FF MUs as compared with the untrained group (the main group effect, Figure 2 and Table 2). As expected, the observed functional changes in trained gastrocnemius muscle were accompanied by a significant increase in triadin content in the medial gastrocnemius muscle (Table 4). On the other hand, we did not find any changes in the concentration of proteins involved in skeletal muscle force potentiation.

Duchateau and Hainaut (1984) were the first who showed that moderate dynamic resistance training induces a paradoxical decrease in the evoked twitch force as well as contraction and half-relaxation times. This was accompanied by an increase in the maximum tetanic tension of human adductor pollicis muscle. In another study, a reduction in the evoked resting twitch torque and twitch:maximum voluntary torque ratio, as well as increase in postactivation potentiation and maximum voluntary contraction torque of elbow flexors following low-intensity resistance training with vascular occlusion were found (Moore et al., 2004). Those results indicate, similarly to our findings, that shortening of the twitch contraction time or even reduction in the amplitude of evoked twitch can coexist with an increase in the twitch potentiation and maximum force capacity of muscle and motor units after the resistance training.

### Resistance Training and FF MUs Twitch Potentiation

The primary mechanism involved in potentiation of twitch force and RFD is skMLCK-mediated phosphorylation of RLC in myofilaments, which enhances sensitivity of the contractile apparatus to Ca^2+^ during contraction (Stull et al., 2011). It is also suggested that PTP results from a transient increase in the number of myosin heads able to interact with the actin as contractile apparatus Ca^2+^ sensitivity increases proportionally to actomyosin ATPase activity (Sweeney and Stull, 1990). There is evidence that overexpression of skMLCK in muscle increases PTP of fast-type IIb muscle fibers (Ryder et al., 2007). However, in the present study, we did not find any change in the muscle levels of either skMLCK or actomyosin ATPase after the training (Table 4).

Twitch force potentiation can coexist with fatigue. For instance, if RLC is phosphorylated, twitch force can be enhanced while high-frequency contractile response is concomitantly depressed (Rassier and Macintosh, 2000). Moderate fatigue caused by prior activity may depress the isometric contractile response by decreasing sensitivity to Ca^2+^ (Westerblad et al., 1991), which may result from elevated levels of metabolites, such as inorganic phosphate (Cooke et al., 1988). After the employed resistance training, we observed that FF units exhibited less decrease in force of 150 Hz tetanus during conditioning stimulation. Previously, we noted that FF MUs were able to better maintain the isometric force of submaximal tetanic contraction at the early stage of the fatigue test (Łochyński et al., 2016) when phosphagens are the main source of energy (Baker et al., 2010). Perhaps muscle fibers of FF MUs become less sensitive to the fatiguing influences of phosphagen breakdown due to preceding conditioning stimulation and well-preserved Ca^2+^ sensitivity of the contractile apparatus. Therefore, both twitch and submaximal tetanic force potentiation was increased after the training.

### Resistance Training and FR MUs Twitch Potentiation

After the weight-lifting training, the twitch contraction time of FR MUs was decreased. The absolute twitch amplitude did not increase, but maximum force was greater after the training (Łochyński et al., 2016), resulting in reduced twitch:tetanus force ratio. These contractile adjustments resembled modifications in twitch mechanical parameters seen after acute administration of dantrolene sodium in rat gastrocnemius muscle (Leslie and Part, 1981). In this condition, Ca^2+^ release is inhibited, but its reuptake (Desmedt and Hainaut, 1977) or actin-myosin cross-bridge cycling is not affected (Takauji and Nagai, 1977). Therefore, in the trained rats, activation of FR MUs in response to a single pulse appeared to be less complete than in the untrained animals. Despite these changes, PTP of FR MUs was retained but not increased after the training (although the PTP ratio presented in Table 3 was increased after training, such comparisons are much less convenient, see Table 2 and Figure 2). This is not surprising as decreasing the twitch:tetanus ratio by limiting initial release of Ca^2+^ from the SR can restore potentiation in conditions that severely limit this ability (Rassier et al., 1997). Therefore, very substantial decreases in twitch duration and reduction in the twitch:tetanus ratio probably rendered FR units more susceptible to potentiation.

Increased slopes of regression expressing the relationship between the twitch contraction time and twitch:tetanus force ratio and PTP capacity of FR MUs observed in the present study seem to be indirect confirmation that this might be the case. In trained animals, not only were the twitch contraction time and twitch:tetanus force ratios reduced, but also these relationships became steeper. That is, MUs with very short contraction times and low ratios became even more susceptible to potentiation than in untrained animals. Furthermore, in response to repetitive submaximal 40-Hz tetanic stimulation, when the amount of Ca^2+^ released to myoplasm is muchly increased with respect to a single stimulus (Baylor and Hollingworth, 2012), enhanced force potentiation was noted in response to the conditioning stimulation after the training (Figure 4B). It is difficult to speculate what caused these specific contractile responses after the resistance training. We found that muscle did not contain more skMLCK, which would suggest an increase in RLC phosphorylation and crossbridge kinetics. Therefore, the underlying cellular mechanism of these specific changes remains to be elucidated.

### Calcium Handling Proteins After the Resistance Training

It is shown that a single bout of resistance exercise results in phosphorylation of RyR1, which makes this channel leaky to Ca^2+^ and disturbs the intracellular homeostasis of this ion (Gehlert et al., 2012). Results of the present study show that muscle concentration of RyR1 protein was not decreased in MG (Table 4) even though it would be a potential mechanism to decrease Ca^2+^ release. The performed training exerted different impact on the junctin and triadin, which are two proteins anchoring calsequestrin with RyR1 to control Ca^2+^ release from the RyR (Beard et al., 2009). We have found that 5 weeks of resistance training resulted in no changes in junctin level, whereas it increased significantly triadin content in the medial gastrocnemius muscle (Table 4). Triadin is a transmembrane glycoprotein of the junctional SR, which is responsible for maintaining excitation-contraction coupling (Wei et al., 2009). Although the exact role of triadin in SR Ca^2+^ release is still elusive (Beard et al., 2009), experimental triadin ablation induces chronic resting elevation of Ca^2+^, probably increasing basal RyR1 activity (Eltit et al., 2010). Moreover, its overexpression is associated with the suppression of the depolarization-induced SR Ca^2+^ release, suggesting inhibition of RyR1 channels in activated skeletal muscle (Rezgui et al., 2005). During muscle contraction, force of muscle fibers of MUs is directly dependent on Ca^2+^ levels in the cytoplasm (Baylor and Hollingworth, 2012). The activity of RyR1 is regulated by its interaction with calcineurin and calstabin molecules, known as FKBP12 or FK506 binding protein (Shin et al., 2002). Triadin can affect FKBP12/RyR1 interaction (Eltit et al., 2010). Association of FKBP12 with RyR inhibits Ca^2+^ release, and dissociation causes opening of the channel complex and intracellular Ca^2+^ release in muscle cells (Gehlert et al., 2015). Although the level of FKBP12 was not altered in our study, still an open question remains how increased triadin concentration affects its control over RyR activity after the resistance training. However, based on previous reports, it seems that an increase in muscle triadin concentration can inhibit SR Ca^2+^ release. This could be a potential mechanism underlying the decrease of the twitch contraction time, and lack of increase (or even reduction) in its amplitude observed here and in previous studies after the resistance training (Duchateau and Hainaut, 1984; Moore et al., 2004; Łochyński et al., 2016).

It needs to be mentioned that we found no changes in SERCA1 content after the training, which is specific to the type II muscle fibers. This suggests that shortening of the potentiated twitch contraction observed in fast MUs after training was not mediated by modifications in sequestration of Ca^2+^ by the SR (Hunter et al., 1999).

### Limitations

With the current technique, the recordings of contractile activity of different MUs cannot be made simultaneously. Therefore, in this study, the phosphorylation levels of RLC before and after the conditioning activation could not be measured. Thus, the knowledge of how much resistance training increases phosphorylation levels and potential mechanisms explaining the observed differences in MU PTP response could not be revealed. Also, the concentrations of studied proteins were measured in the whole muscle. Therefore, it was impossible to determine the expression of these molecules in muscle fiber samples belonging to specific MU phenotypes. Future studies should take this into account and also assess if resistance training affects SR Ca^2+^ release and myoplasmic accumulation during potentiated non-potentiated contractions and Ca^2+^ sensitivity of fast muscle fibers. It must be also noted that extrapolation of our findings from rats to humans is limited despite some similarities in the physiological and anatomical properties of MUs in both species of mammals.

### Possible Functional Consequences

Previous studies provide evidence that high-load exercises are much more effective than light-load exercises in inducing muscle potentiation (Esformes et al., 2010). Perhaps this is because neural commands to muscle fibers control myosin RLC phosphorylation and muscle potentiation. It is postulated that, during forceful muscle contractions, high-frequency rates of MU firing are evoked (Sale, 2002). Based on relationship between the MU’s size and firing rates (Duchateau et al., 2006), it can be assumed that, during the course of the used voluntary resistance training with progressively increasing load (70–85% 1 RM), FR MUs were activated with much higher firing rates and generated stronger contractions than FF units. On the other hand, based on time of daily activity (Hennig and Lømo, 1985), the recruited FF units probably increased their total activity time much more than FR units. These could be underlying causes of differences in contractile adjustments and potentiation ability during single and repetitive stimulation in both MU types.

Summarizing, the ability of fast MUs to potentiate twitch force after the short-term resistance training seems to depend on training-related contractile adjustments. Substantial decrease in the twitch contraction duration results in reduction of twitch:tetanus force ratio but probably also renders FR units more susceptible to potentiation. As a result, force potentiation evoked by a single stimulus is not fully expressed, but it is substantially increased during repetitive submaximal tetanic stimulation. On the other hand, non-potentiated twitch parameters are not affected in FF MUs after the training. These units are less prone to fatigue in response to repetitive tetanic stimulation. Thus, they seem to increase force potentiation predominantly by decrease in fatigability. Further work is warranted to establish if the increase in muscle triadin concentration is connected with the decrease in Ca^2+^ release from the SR or has any impact on the reduction of twitch duration and force after the resistance training. These findings are important for better understanding contractile adjustments after resistance training.

### Perspectives

Joint injury, bone fracture, or detraining may result in disuse atrophy. In atrophied muscle, activation of the contractile proteins with a single pulse is more complete, which limits the contraction range in which potentiation mechanisms can effectively enhance the twitch force (Wei et al., 2009). We show that resistance training decreases twitch activation level in FR MUs composed predominantly of type 2a/x muscle fibers. In this way, training may contribute to restoration of potentiation sensitivity in the atrophied fast-type muscle. Muscle potentiation capacity depends on the level of fatigue, which lingers after preceding contractile conditioning. If rest time is too short, there may be no improvement in contractile performance (Chiu et al., 2003). However, if too much rest is allowed, potentiation may no longer persist. This so-called “window of opportunity” (Docherty and Hodgson, 2007) is of great importance for practical utilization of muscle potentiation in sports performance and rehabilitation. In resistance-trained athletes, force potentiation appears to be higher and expressed earlier than in recreationally active individuals (Chiu et al., 2003). Perhaps, because fast MUs can augment force more (Figure 4C), are less prone to fatigue after conditioning contractions, and able to maintain potentiation capacity during initial 10–15 s of submaximal fatiguing activity (Figure 4D), the window of opportunity is wider after the resistance training.

## Data Availability Statement

The datasets generated for this study are available on request to the corresponding author.

## Ethics Statement

The animal study was reviewed and approved by the Local Ethical Committee for Animal Experiments, Poznań University of Life Sciences, Poland.

## Author Contributions

DŁ, DK, and JC conceived and designed the research and conducted the data interpretation and analyses. DŁ wrote first draft of the manuscript. DŁ, DK, MG, JM, TP, and JC performed the experiments and reviewed the manuscript submitted for publication. All authors revised and approved the final version of the manuscript.

## Conflict of Interest

The authors declare that the research was conducted in the absence of any commercial or financial relationships that could be construed as a potential conflict of interest.
